# Continuous gait cycle index estimation for electrical stimulation assisted foot drop correction

**DOI:** 10.1186/1743-0003-11-118

**Published:** 2014-08-09

**Authors:** Christine Azevedo Coste, Jovana Jovic, Roger Pissard-Gibollet, Jérôme Froger

**Affiliations:** DEMAR INRIA/LIRMM, UM2, CNRS, Montpellier, France; INRIA, Montbonnot, 1, France; CHU Nîmes, Nîmes, France

**Keywords:** Post-stroke, Foot drop, FES, Gait cycle index (GCI)

## Abstract

**Background:**

Walking impairment after stroke can be addressed with the use of drop foot stimulators (DFS). Many studies have demonstrated that DFS improves walking speed, reduces spasticity and reduces the physiologic effort of walking. Current DFS, through activation of the common peroneal nerve, elicit ankle dorsiflexion during swing phase of gait. DFS are generally piloted by force sensing resistor placed in the shoe of the affected side with stimulation triggered ON by heel rise and triggered OFF by heel strike. A tilt sensor can also be used with stimulation triggered by the tilt of the shank of the affected leg. These triggering approaches are the standard for initiating stimulation. However, the real-time modulation of FES intensity to provide more optimized delivery of stimulation and also to regulate dorsiflexion in the presence of disturbances, such as fatigue and spasticity may increase the number of potential users of DFS. Concerning research domain, stimulators that would allow modulating the stimulation pattern in between heel rise and strike events would allow exploring new stimulation strategies. We propose to extract continuous information: the gait cycle index (GCI), from one inertial measurement unit (IMU) measuring shank tilt angle. In order to illustrate the use of this real-time information, we show the feasibility of piloting an electrical stimulator.

**Methods:**

12 subjects with post-stroke hemiplegia participated. A wireless IMU was placed on the unaffected shank and was used to estimate GCI. Subjects performed 3 trials in each of the 3 conditions: C1 no stimulation aid, C2 electrical stimulation assistance triggered by heel switch, C3 electrical stimulation assistance triggered from GCI.

**Results:**

1) the proposed algorithm was able to real-time estimate GCI, 2) events could be extracted from GCI information in order to trig a DFS.

**Conclusion:**

The estimation of the continuous GCI in individuals with stroke is possible. Events can be extracted from this information in order to trig a stimulator. These results are a first step towards the possibility to investigate new DFS paradigms based on real-time modulation of stimulation parameters.

## Background

Walking impairment after stroke is a common and universal problem [1, 2] that is now being addressed successfully with the use of drop foot stimulators (DFS) [3]. Many studies have demonstrated that a DFS improves walking speed, reduces spasticity, makes walking safer and reduces the physiologic effort of walking for stroke survivors [4–13]. Current dropped foot stimulators, through activation of the common peroneal nerve, elicit ankle dorsiflexion on swing phase of gait. Reflex stimulation of the CPN can also be used to improve hip and knee excursion where there is extensor spasticity necessitating compensatory gait habits such as hip circumduction. Dropped foot stimulators are generally piloted by a force sensing resistor heel switch placed in the shoe of the affected side with stimulation triggered ON by heel rise of the affected foot and triggered OFF by heel strike [3, 6, 14–21]. A tilt sensor can also be used with stimulation triggered by the tilt of the shank of the affected leg [22]. These triggering mechanisms, based on event detection, have proved very reliable and are now the standard for initiating stimulation during the gait cycle in most common indoor and out door walking conditions [23–25].

However, real-time control of stimulation intensity is still not available in existing devices [3]. The modulation of FES intensity to provide more optimized delivery of stimulation and also to regulate dorsiflexion in the presence of disturbances, such as fatigue and spasticity may increase the number of potential users of the technology [26].

It would also be of great importance to be able to analyze the orthotic and clinical outcome of other type of stimulation strategies other than triggering ON and OFF a fixed pattern based on gait events. Some research studies have suggested that improvement in orthotic performance could be achieved using stimulus intensity shapes matching more closely the natural tibialis anterior biphasic activation pattern than the trapezoidal shape classically used in the stimulators [27–31].

In this article we propose to extract continuous tilt angle information from one inertial sensor fixed on patient shank. As a first attempt to use this real-time information, we show the feasibility of triggering an electrical stimulator based this information. We embedded our gait observation algorithm within a system involving a commonly used Odstock stimulator. The goal was to show the feasibility of processing the gait cycle index (GCI) from one sensor, and to use it to pilot the stimulator.

In the present study, we: 1) explore the feasibility of continuous tracking gait cycle in individuals with foot drop using one sensor placed on the unaffected leg shank and 2) validate the feasibility of combining the observation algorithm with a DFS controller.

## Methods

### Subjects

12 subjects (10 males/2 females, age 54 ± 14) with hemiplegia were included. Subject characteristics are given in Table  1. All subjects provided informed consent and the study was conducted in accordance with the principles of the Declaration of Helsinki and was approved by the local Ethics Committee (CPP Nîmes).

**Table 1:** **Sujects’ characteristics**

Included patients characteristics							
Subject ID	Sex/age	Time since stroke (months)	Stroke diagnosis	FAC	BI	Aid	1st stim. condition
1	M/48	94	Left/isch/ST	5/5	95/100	AFO	C3
2	M/54	4	Right/hemo/ST	4/5	75/100	CC + AFO	C2
3	F/48	3	Left/isch/ST	4/5	65/100/100	SC + AFO	C2
4	M/27	62	Left/isch/ST	5/5	100/100	AFO/OS	C3
5	F/49	7	Right/hemo/ST	5/5	80/100	OS	C2
6	M/82	86	Right/isch/ST	4/5	75/100	SC + OS	C2
7	M/48	42	Right/isch/ST	4/5	70/100	TC + OS	C2
8	M/48	4	Left/isch/ST	2/5	60/100	CC + AFO	C2
9	M/63	2	Left/isch/ST	4/5	75/100	CC	C2
10	M/60	34	Left/hemo/ST	5/5	95/100	-	C3
11	M/59	102	Left/isch/ST	4/5	75/100	CC + OS	C3
12	M/59	22	Left/isch/ST	4/5	80/11	CC + OS	C3

With: **Stroke diagnosis**: ischemic (isch)/hemorrhagic (hemo)/infra-tentional (IT)/supra-tentorial (ST). **FAC:** Functional ambulation Categories (Collen et al., [23]). **BI:** Barthel index assessment of daily activity impairment (autonomy evaluations). OS: orthopaedic shoes. - **SC:** simple cane- **CC:** Canadian crutch- **TC:** tripod cane.

### Observation algorithm for gait phases estimation

The algorithm was first introduced and described by Heliot and Espiau in [32], where more technical details can be found. The method allows estimating the phase of the gait cycle of a healthy person using a micro-sensor, which associates 3 accelerometers and 3 magnetometers, placed on person’s thigh. The method was designed to allow humanoid robot to mimic the walk of a human demonstrator [32]. Ahn et al., based on a similar framework, have proposed a walking model, which can reproduce some behaviors of human walking [33]. But this approach is not intended to be used online for human motion tracking and has not been validated experimentally.

Our algorithm is based on defining OFFLINE a model of a system, which in our case is human gait. In the field of control of locomotion in humanoid robots, the bipedal gait has been modeled as a non-linear oscillator [32, 34]. Two common oscillators can be used for this purpose: the van der Pol oscillator and the Rayleigh oscillator, which are very similar. The Van der Pol oscillator was used in [32], and experimentally proved to be robust and suitable for human gait modeling. Therefore, the chosen model in this study is Van der Pol oscillator which details are given in [35]. Briefly, the Van der Pol oscillator is a self-oscillating system that has a stable periodic solution with a period of T_0_ and a frequency of ω_0_. The equation of Van der Pol oscillator used in this study is given by (1).
1

where μ, and ω_0_ are positive constants. The choice of μ, ω_0_, and b constants is explained bellow.

**Figure 1:**
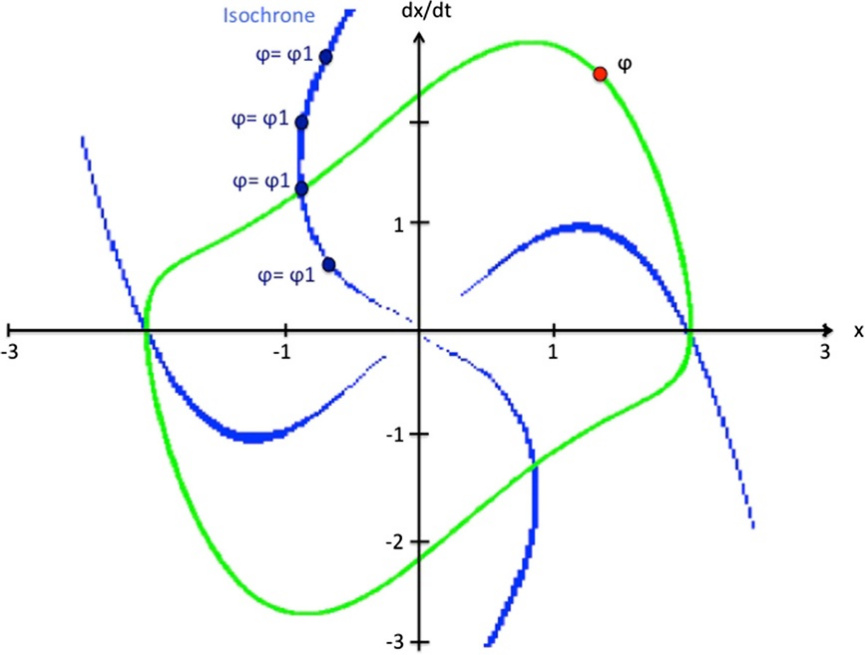
**Example of a limit cycle of Van der Pol oscillator and isochrones curves in the phase plane for μ = 1 , ω**_**0**_ **= pi/2 , and b = 0.**

In the phase plane (position versus velocity), periodic stable solution of the oscillator is a trajectory called a limit cycle (Figure 1). The phase φ is a coordinate along this limit cycle:

2

The phase grows uniformly in the direction of motion and gains 2π at each rotation. There is a need to define the φ variable in such a way that it rotates uniformly in the cycle of the oscillator. We therefore define OFFLINE an isochron matrix that allows us to associate a phase value to any point in the vicinity of the limit cycle. Each point in the vicinity of the limit cycle is associated with a point in the limit cycle (Figure 1). This makes it possible to compute a phase φ even in the vicinity of the limit cycle, for example when the observed behavior differs from that of the reference.

Based on control theory framework, it is theoretically possible to build an observer of this system that will be able to estimate the internal state of the system, in our case gait of a person with drop foot modeled as Van der Pol oscillator, from the system output measurement. In control theory, a state observer is defined as a system that provides an estimate of the internal state of a given real system, from measurements of the input and output of the real system [34], and could be represented using the following set of equations:
3

where: y is the output of the system, and x1, and x2 are the states of the system which should be estimated. In our case, y corresponds to a measurement of shank angle of the unaffected leg. The shank angle was chosen because it is the easiest to be obtained by the sensor technology used in this study. Knowing the value of y, and solving the system of equations (3), we can obtain state variables x1 and x2. From the observer’s state variables we can compute the phase φ of the oscillator. We defined the gait cycle index GCI from φ normalization in order to get values ranging from 0 and 100% (the cycle starts and stops at unaffected leg heel strike):
4

**Figure 2:**
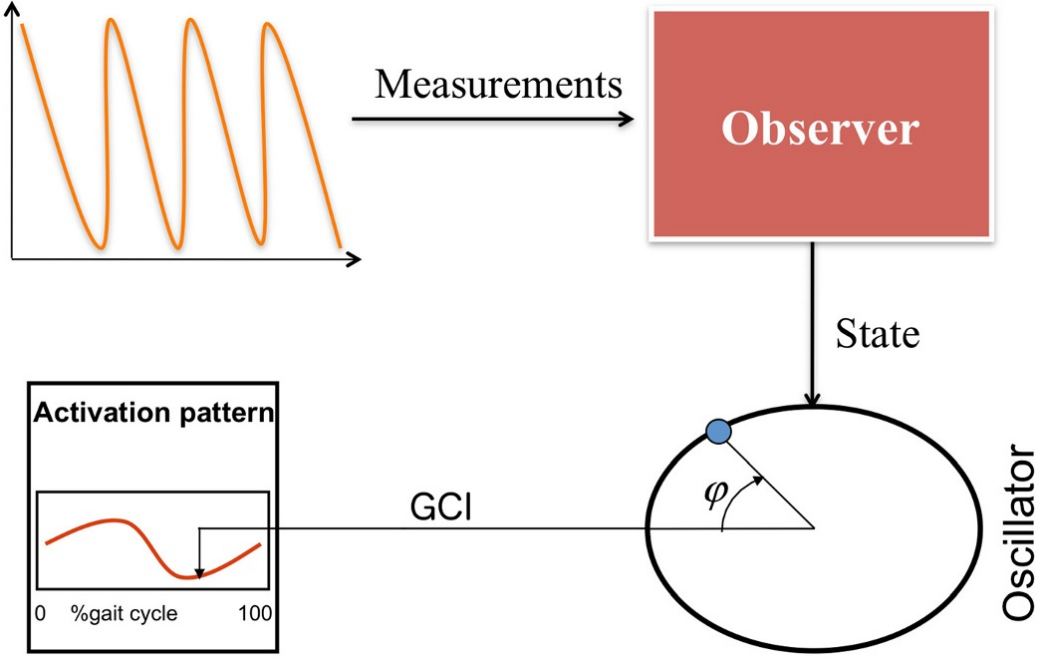
**Principle of the observation algorithm.** Inertial measurement unit (IMU) is used to estimate unaffected leg shank angle. The measurement of the shank angle is set as an input to the state observer that estimates the variable φ. The gait cycle index (GCI) is calculated from variable φ and used to trigger the electrical stimulator.

The principle of described algorithm is given in Figure 2.

The last step is defending the values of μ, ω_0_, and b constants in (1) that would fit the sensor measurement into this oscillator model. This problem can be solved by using dynamic optimization technique called Feasible Sequential Programming Technique (SQP) [36]. SQP technique finds the values of μ, ω_0_, and b constants that minimize a quadratic function of the error between measurement and output of our model. The mathematical details about the calculations are given in [37]. Prior to the experiments the participant was asked to walk a couple of steps. The measurement of unaffected shank angle was used to identify oscillator parameters.

**Figure 3:**
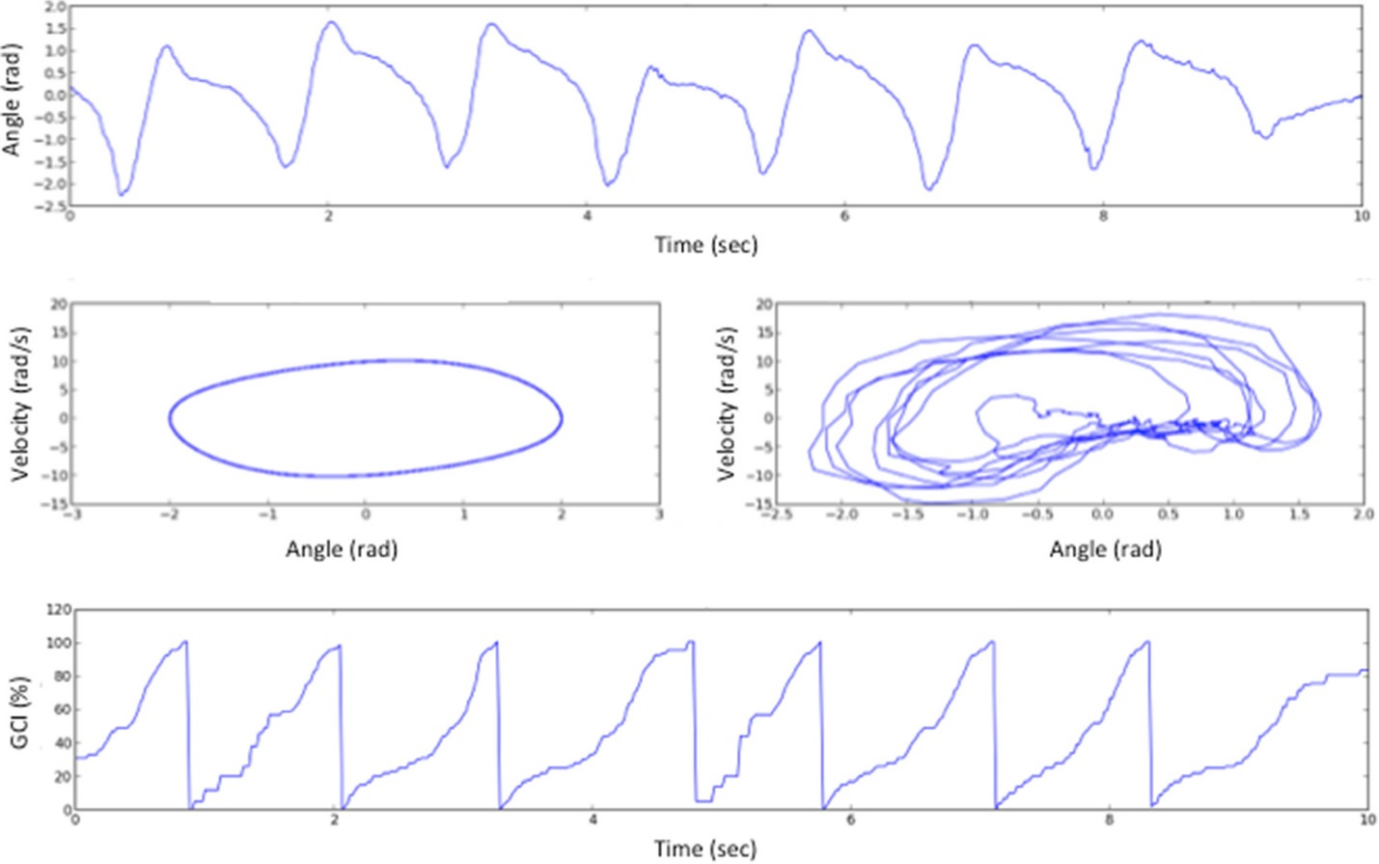
**From shank angle observation to GCI estimation.** Top: Measured shank angle (online). Middle left: Van der Pol model of the system (offline). Middle right: Phase portrait of the measured angle evolution (online). Bottom: Estimation of the Gait Cycle Index.

We summarize the different steps of the method (see Figures 2 and 3).

- Oscillator design (OFFLINE)

1. The subject is asked to walk a few steps during which the shank angle is measured.
2. The oscillator parameters μ, ω_0_, and b are identified through a dynamic optimization problem: the error between the measured shank angle and the oscillator output is minimized. The isochron matrix is computed.
3. The oscillator parameters are updated in the observer of the system.

Gait Cycle Index estimation (ONLINE) - the observer computes φ and GCI variables in real-time based on the measured shank angle y.

### Equipment

**Figure 4:**
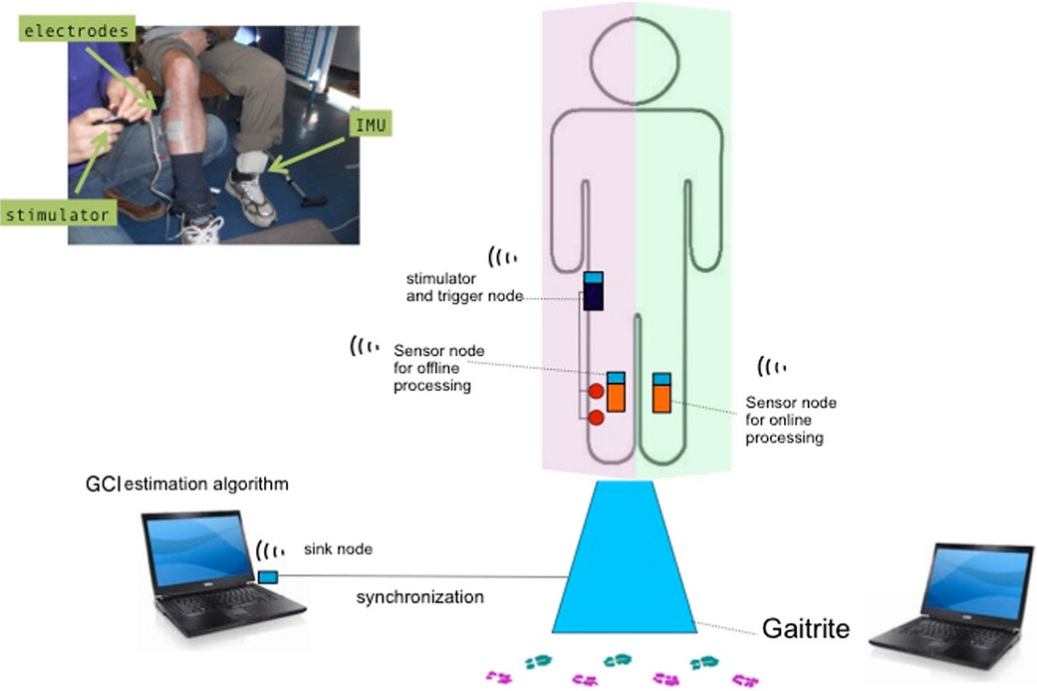
**System architecture.** Description of the system architecture used in the study. A sensor node (inertial measurement unit (IMU)) is placed on the unaffected side shank. Data is sent to the sink node of the laptop. Data is processed on the laptop and a gait cycle index is estimated. Depending on the GCI value, the stimulator is switched ON through its trigger node. An extra sensor node also sends data to the sink node and the data is saved for offline processing.

The developed system is based on a wireless architecture of sensors and actuators using WSN430 technology (https://www.iot-lab.info/). A WSN430 node is an electronic system which insures 3 functions: data acquisition using daughter board sensor-specific, data processing based on a micro-controller (Texas Instruments MSP430) and wireless radio- frequency communication (based on Texas Instruments CC1100). In our architecture, 3 types of WSN430 nodes are used: one sink node connected to a laptop via a serial port, one sensor node placed on the participants’ lower limbs, and one control node which triggers the stimulator as shown in Figure 4. This node allows communication with the network of sensor node and actuator node.

#### ONLINE GCI computation

One inertial sensor node is placed on the patient’s unaffected shank (Figure 4). It integrates one 3-axis accelerometer (STMicroelectronics LIS3LV02DQ) and one 3-axes magnetometer (Honeywell HMC5843). The sensor node sends data to the laptop via the sink node. The sampling frequency was set at 100 Hz. The shank angle is then computed based on accelerometer and magnetometer signals as described in [26]. This angle y is used by the Observation algorithm for gait phases estimation, described in section 1.2, to estimate the oscillator state variables and then compute the gait cycle index GCI. The algorithm is run on the laptop (Linux/Python). The sink node also serves to send data to the actuator nodes.

#### Stimulator

**Figure 5:**
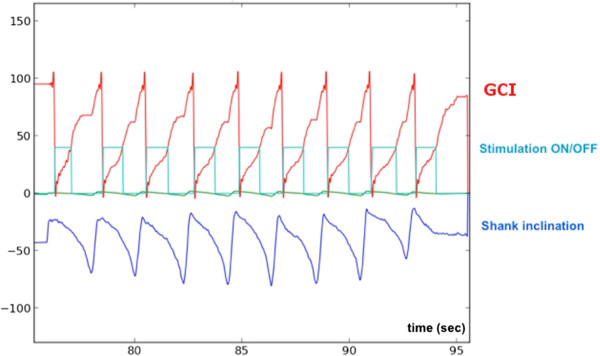
**Illustration of the estimation/triggering algorithm.** The shank inclination measured from the inertial sensor allows to estimate the gait cycle index online and to modify the stimulator switch input depending on the GCI values (here stimulation is ON for GCI values between 0 and 40%).

A single-channel Odstock Dropped Foot Stimulator was used. Two skin surface electrodes were placed, over the common peroneal nerve and at the motor point of the tibialis anterior. The stimulator parameters (current intensity and pulse width) are triggered offline on the patient in order to obtain efficient dorsiflexion/eversion movement without discomfort or pain. The stimulator can be used in “normal” mode using a footswitch placed under the affected side heel in order to switch the stimulation ON at heel off and OFF at heel strike. To test the algorithm described in the “Observation algorithm for gait phases estimation”, the actuator node was plugged onto the switch connector of the stimulator in order to trigger ON/OFF the stimulation depending on the information received from the sink node (Figure 5).

### Protocol

In the protocol, each subject walked along the GAITRite walkway. Each subject performed 3 successive trials for each of the 3 conditions: C1) No stimulation, C2) Stimulation triggered on the basis of heel switch information, C3) stimulation triggered on the basis of the GCI information. C2 and C3 were applied in a randomized order among the patients (see Table 1).

Condition C2 was used as a control condition for stimulation. In this preliminary validation work, the goal was to reproduce the behavior of heel switch triggering using our experimental observation algorithm. As the swing phase in normal human walking lasts 40% of the gait, the stimulation was triggered for 0 ≤ GCI ≤ 40% for almost all the subjects in C3 condition. The exceptions will be discussed here after.

### Statistics

Based on the GAITRite information, the data recorded during the trials were slip into strides.

- The validation of the GCI estimation algorithm was done on the C3 condition. To assess variability between strides, we computed the mean value of the cross-correlation between the GCI variable waveform over the strides of each patient. Similarly, we applied this to shank inclination waveform. To assess the validity of the GCI information, we computed the standard deviation of the GCI values corresponding to heel OFF and heel ON (heel strike) events extracted from GAITRite software.
- A t-test was carried out to compare the average walking speed in each condition (C1, C2, and C3).

## Results

Table 2 shows, for each subject, the number of trials during which the stimulator was not triggered in affected leg heel stride moment, as programmed by the proposed GCI estimation algorithm. Table 2 also gives the number of performed trials for each subject.

**Figure 6:**
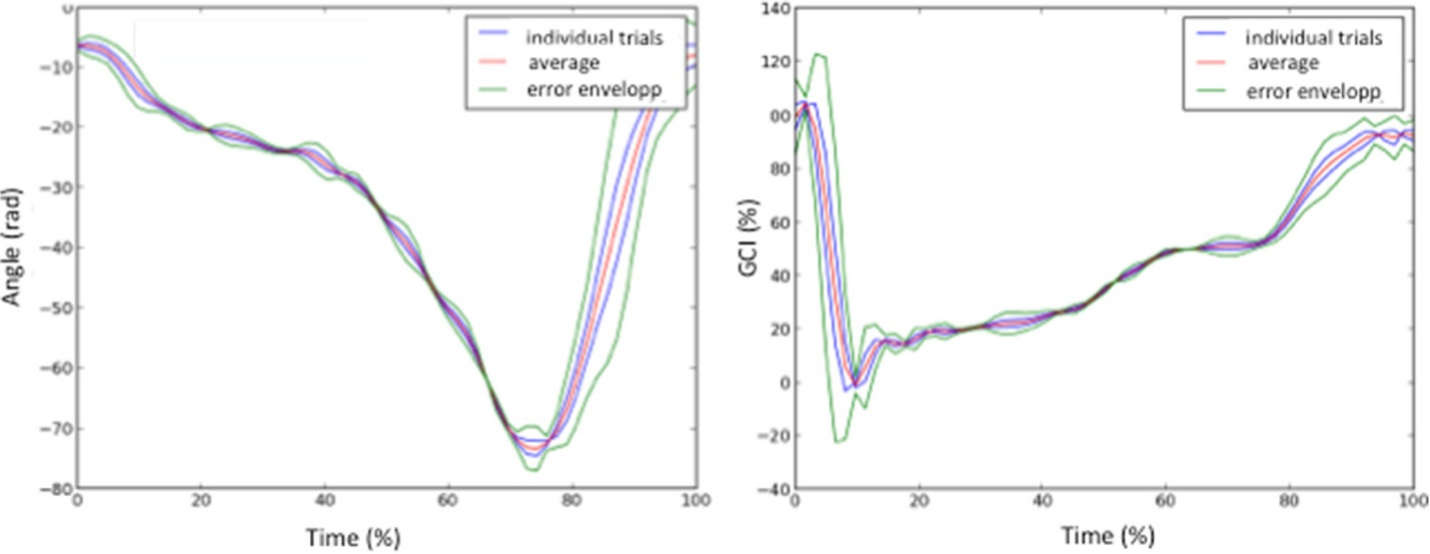
**Example of the repeatability of shank inclination and GCI information for each placed on the unaffected side shank.** It is segmented using GAITRite data. One stride corresponds to the time between two heel offs of the (HOFF) events of the affected leg. Similarly, the estimated GCI for each stride of one trial is plotted.

In Figure 6 correlation coefficients between each GCI signal computed for each individual heel stride for each trial in the C3 condition are shown. The average correlation coefficient was 0.74 ± 0.13. We also analyzed, for each trial, the correlation coefficient between each unaffected leg shank inclination signal estimated for each individual stride in the C3 condition. The results are shown in Figure 6. The average correlation coefficient obtained was 0.76 ± 0.17

**Table 2:** **Ability of the proposed system to trigger the stimulator**

ID	Number of analyzed strides	Communication errors inducing incorrect triggering
1	14	0
2	18	1
3	24	0
4	14	0
5	30	4
6	12	0
7	34	6
8	35	0
9	18	0
10	20	0
11	26	0
12	37	1

**Figure 7:**
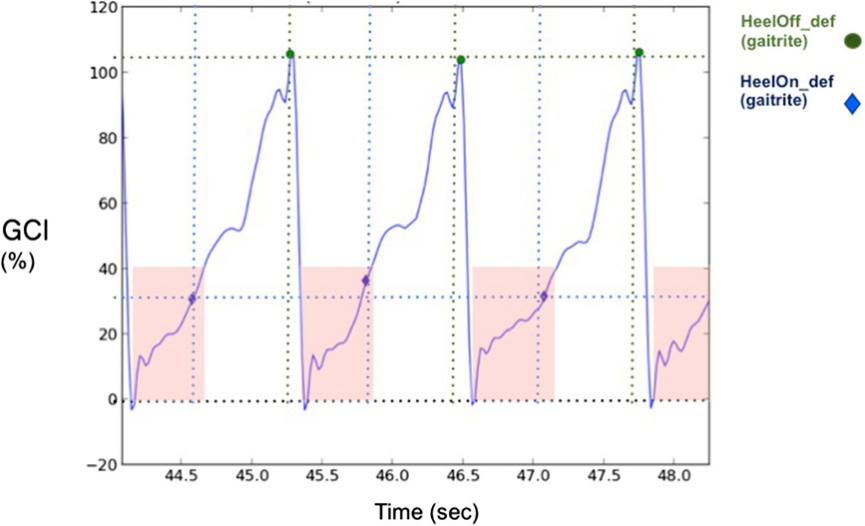
**Comparison of the GCI estimated and the GAITRite information.** We compare the values of the GCI corresponding to given events of the gait cycle affected leg heel off and heel strike obtained from GAITRite. In light red, the intervals for which the GCI ranged from 0 to 40%.

The GAITRite walkway was used as a gold standard to verify heel on and heel off moments of affected leg for each trial of each subject. The GCI values computed in the C3 condition, corresponding to Heel OFF and Heel ON were compared with events extracted from GAITRite data for the affected leg. The comparison between heel ON and heel OFF instants estimated using GAITRite system and proposed Observation algorithm for gait phases estimation is shown in Table 3 and an example is given in Figure 7.

**Figure 8:**
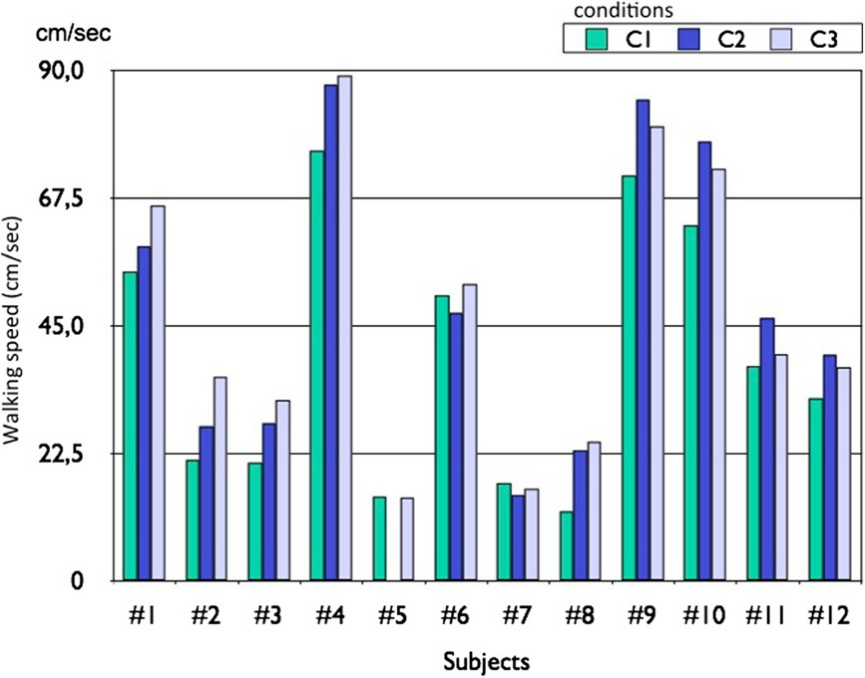
**Comparison of walking speed in the 3 conditions.** We compare the values of the walking speed in C1 (no stimulation), C2 (footswitch based stimulation) and C3 (GCI estimation algorithm based stimulation).

In Figure 8, we show the influence of the different conditions on the walking speed. On average, the walking speed was 12.8% higher in C3 (GCI estimation algorithm based stimulation) than in C1 (no stimulation), this difference was statistically significant (t-test, p < 0.05). No statistically significant statistical difference between the walking speeds in condition C2 (footswitch based stimulation) compared with walking speeds in condition C3 was observed.

**Table 3:** **Variability of the GCI information**

	GCI value (%) corresponding to Heel OFF event*		GCI value (%) corresponding to Heel ON event*	
ID	Mean	Std	Mean	Std
1	32.8%	2.3%	94.0%	5.4%
2	44.5%	5.4%	88.2%	20.4%
3	57.9%	6.3%	87.0%	19.5%
4	37.0%	4.9%	92.9%	16.2%
5	70.8%	1.6%	94.0%	13.6%
6	46.2%	4.6%	90.7%	15.7%
7	65.0%	5.3%	23.8%	19.1%
8	43.3%	11.8%	12.4%	11.7%
9	33.3%	4.9%	77.6%	13.6%
10	44.3%	5.0%	92.6%	6.3%
11	42.2%	4.6%	90.9%	22.0%
12	45.7%	18.6%	91.1%	15.7%

*Extracted from GAITRite.

## Discussion

### Ability of the observation algorithm to compute the gait cycle index GCI

In this study we used the observation of the unaffected leg shank angle to detect the heel strike moment instead of commonly used heel-floor contact. Strong positive correlation between unaffected leg shank inclinations demonstrates that the shank angle is a variable, which has similar repeatable shape during the gait of a person with drop foot, and could be potentially used to trigger the electrical stimulator.

In order to validate the relevance of the GCI information, we compared the GCI values computed in the C3 condition, corresponding to Heel OFF and Heel ON events extracted from GAITRite data for the affected leg (Figure 7). The aim was to determine the extent to which the GCI variable varies from one stride to the next (Table 3) and if a given GCI value could reliably represent a fixed gait event. Even though the oscillator parameters should ideally be identified on a reference trajectory from a gait event after the patient is stimulated and not on the C1 condition, as was done, the results show that the GCI variable was stable over the strides. The GCI information is more reliable when the standard deviation (Std) is low. The mean Std for all patients was 6.3% of the GCI for Heel OFF, which roughly corresponds (assuming that the GCI evolution is linear) to 0.13 s and 15% of GCI for Heel ON, which corresponds (assuming that the GCI evolution is linear) to 0.3 s. The maximum standard deviation was 22% (patient 11), which corresponds (assuming that the GCI evolution is linear) to 0.44 sec (half a step). From a mathematical point of view, the performances are good (<15%). From a functional point of view the corresponding error in seconds compared with the duration of gait cycle of a person with drop foot would need to be improved in order to allow for a fine triggering of the stimulation. These averaged performances will have to be improved in some patients in order to allow triggering a stimulator at any instant of the gait cycle. This should be possible by identifying the reference model parameters after an adaptation period of the patient to stimulation.

From the Table 3, we can observe low heel strike coefficient of variations for subject 7 and subject 8. Those subjects’ walk was more impaired compared to other subjects. This makes general comments difficult on the performance of GCI over all the subjects who have participated in this study.

### Stimulation triggering

Among all 12 subjects, 282 strides were analyzed for the trials in the C3 condition. In only 4% of the cases, the stimulation was not triggered in affected leg heel strike moment, as expected. That was due to problems with the wireless communication such as loss of wireless signal (Table 2). For patient 5 it was impossible to test the C2 condition. Indeed, we could not use a footswitch under the affected side heel neither the unaffected side toes. Indeed, the gait of this patient was too impaired and the foot strikes too lateral to make a direct link between the footswitch state and the stimulator ON/OFF status. However, it was possible to test the C3 condition for this same patient. In this case, stimulation was ON for GCI^2^ ranging from 30% to 70%. Patients 1 to 4 and 7 to 12 were stimulated for GCI values ranging from 0% to 40%. Indeed, for these patients the swing phase duration was similar to normal gait. Patient 6 was stimulated for GCI values ranging from 0% to 50%.

The proposed approach allows selecting instants in between heel OFF and heel ON in order to appropriately trigger stimulation ON or OFF. This opens the possibility of exploring new strategies for stimulation like starting stimulation before heel OFF or applying biphasic stimulation in between heel OFF and heel ON. Compared to other approaches the optimization of oscillator parameters to define the reference model for each patient takes several minutes but can be an automatized procedure, which would be done once by the clinician. The use of this system by a patient is not more constraining than existing ones.

## Conclusion

This article experimentally validated, on post stroke hemiplegic individuals, a new observation algorithm for gait phase estimation. Gait cycle index is estimated online from a single wireless sensor placed on the lower limbs. GCI corresponds to a percentage of individual gait cycle completion. Despite the heterogeneity in patient characteristics, in of 95.7% the trials the algorithm was able to compute GCI and trigger the electrical stimulator in affected leg heel strike moment.

The main limitation of the study is an imprecise detection of heel ON and OFF events when compared with the detection of the same events using GaitRite system. The algorithm performances will have to be improved in order to decrease the standard deviation of events extracted from GCI. This should be possible by identifying the reference model parameters after some adaptation period of the patient to stimulation.

The GCI is associated with the rhythmic nature of the gait and allows us to track the gait cycle independently of the motion amplitude or temporal aspects. The GCI is related to the limit cycle of the oscillator used to model the gait, associated with isochrone curves. Intrinsically, due to the mathematical framework used, our method is robust to gait changes, which is not the case when using fixed events.

In this study, we showed the feasibility of triggering an electrical stimulator based on the events extracted from the GCI. We embedded the Observation algorithm for gait phases estimation within a system involving a commonly used Odstock stimulator. The goal was to show the feasibility of processing the gait cycle index from one sensor, and to use it to pilot the stimulator. In order to, potentially, improve the FES assisted clinical performances and incorporate the advantages of our approach, we plan to use a programmable stimulator. Indeed, by online tracking of the continuous evolution of the gait cycle index, it could be possible to predict gait events and adapt the stimulation parameters and stimulation time.

By using a programmable stimulator we could explore the clinical interest of stimulating at different instants of the gait cycle, which is not possible with other existing methods. The method proposed here could also be used in case of walking on uneven ground terrain, such as stairs. The proposed method could also be used in the case of strong foot inversion and eversion when heel-floor contact cannot be detected using the footswitch technology.

The main contribution of this study in the field of bio-medical engineering is the potential to explore new stimulation strategies that may have clinical validity. In the future we plan to study the influence of the timing of stimulation onset and adaptive biphasic stimulation and its effect on orthotic performances/outcomes (walking speed, propulsion force, etc.). We will also develop new controllers with auto-adaptive properties in order to automatically modulate stimulation parameters in order to assess walking in more challenging conditions (stairs, slope…).

## Abbreviations

FES: Functional electrical stimulation
DFS: Droop foot stimulator
GCI: Gate cycle index
Std: Standard deviation.
